# Molecular Mechanisms of mtDNA-Mediated Inflammation

**DOI:** 10.3390/cells10112898

**Published:** 2021-10-26

**Authors:** Anna De Gaetano, Kateryna Solodka, Giada Zanini, Valentina Selleri, Anna Vittoria Mattioli, Milena Nasi, Marcello Pinti

**Affiliations:** 1 Department of Life Sciences, University of Modena and Reggio Emilia, 41125 Modena, Italy; anna.degaetano@unimore.it (A.D.G.); kateryna.solodka@unimore.it (K.S.); giada.zanini@unimore.it (G.Z.); valentina.selleri@unimore.it (V.S.); 2 National Institute for Cardiovascular Research-INRC, 40126 Bologna, Italy; vittoria@unimore.it; 3 Department of Surgery, Medicine, Dentistry and Morphological Sciences, University of Modena and Reggio Emilia, 41125 Modena, Italy; milena.nasi@unimore.it

**Keywords:** mtDNA, mitochondria, extracellular cf-mtDNA, STING, TLR9, inflammasome

## Abstract

Besides their role in cell metabolism, mitochondria display many other functions. Mitochondrial DNA (mtDNA), the own genome of the organelle, plays an important role in modulating the inflammatory immune response. When released from the mitochondrion to the cytosol, mtDNA is recognized by cGAS, a cGAMP which activates a pathway leading to enhanced expression of type I interferons, and by NLRP3 inflammasome, which promotes the activation of pro-inflammatory cytokines Interleukin-1beta and Interleukin-18. Furthermore, mtDNA can be bound by Toll-like receptor 9 in the endosome and activate a pathway that ultimately leads to the expression of pro-inflammatory cytokines. mtDNA is released in the extracellular space in different forms (free DNA, protein-bound DNA fragments) either as free circulating molecules or encapsulated in extracellular vesicles. In this review, we discussed the latest findings concerning the molecular mechanisms that regulate the release of mtDNA from mitochondria, and the mechanisms that connect mtDNA misplacement to the activation of inflammation in different pathophysiological conditions.

## 1. Introduction

Mitochondria are traditionally defined as the “powerhouse of the cell”, as their primary role is to produce ATP that fuel cell functions by means of oxidative phosphorylation (OXPHOS). However, this definition is extremely limitative, as it does not include the plethora of metabolic and non-metabolic functions played by mitochondria within the cell. Just to mention a few, mitochondria regulate apoptosis, are involved in heme and cholesterol biosynthesis, participate in cell calcium sequestration and release, and are the main source of reactive oxygen species (ROS) in the cell.

In the last years, mitochondria have also been center stage in the field of immunology, for at least two reasons. From one side, an increasing body of evidence has clarified that metabolic reprogramming of the immune cells is crucial for a proper regulation of immune activation, and that the regulation of mitochondrial metabolic pathways (respiration, beta oxidation, and OXPHOS) is used to modify immune cell properties and shape the immune response [1,2]. From the other side, it has been demonstrated that mitochondria do play a direct role in the activation of the immune response, as different mitochondrial products, when released from the organelle, can directly trigger an innate immune response [3]. Among these mitochondrial products, mitochondrial DNA (mtDNA), the own organelle genome, has been widely studied as danger signal, i.e., as a molecule that gives a signal of alarm to the organism and activates an inflammatory program in target cells.

In this review, we critically discuss the most recent observations that link mtDNA with innate immunity, paying particular attention to the molecular mechanisms that link release of mtDNA in the bloodstream, mtDNA binding, and activation of the immune response.

## 2. mtDNA as a Danger Signal

### 2.1. The Evolution of the Mitochondrion and Its Inflammatogenic Role

The inflammatogenic role of mitochondrial products, and in particular of mtDNA, is an indirect consequence of the endosymbiotic origin of mitochondria. The “endosymbiotic theory” was proposed and described for the first time by Lynn Margulis in “On the origin of mitosing cells” in 1967 and confirmed by phylogenetic analysis during the following years [4,5,6,7,8]. The endosymbiotic relationship that originated mitochondria derives from the integration of an alpha proteobacterium into an Asgard Archaea occurred ~2 billion years ago [4,9,10,11]. The complex changes occurred during evolution have transformed the autonomous endosymbiotic bacterium into mitochondrion, the specialized intracellular organelle responsible for the generation of the chemical energy in the form of adenosine triphosphate (ATP), and with many other physiological roles in biochemical and metabolic pathways such as apoptosis, calcium homeostasis, and synthesis of organic chemical compounds [4]. Several peculiar features of bacteria have been preserved in mitochondria that make mitochondrial products potentially recognizable by the immune system as “foreign” molecules. This phenomenon, demonstrated for various bacterial proteins and for bacterial genetic material, has been shown also for mtDNA. Human mtDNA is a molecule of 16,569 base pairs (bp) encoding tRNAs, rRNAs, and 13 subunits of the respiratory chain [12,13,14]. mtDNA has kept similarities to bacterial chromosome: it is structured in a circular loop, it lacks histones, and it contains unmethylated CpG motifs [12,15]. It is organized in nucleoprotein structures, named nucleoids, whose packaging is maintained by the mitochondrial transcription factor A (TFAM). mtDNA and other mitochondrial components have been described as damage-associated molecular patterns (DAMPs), similarly to exogenous microbial products known as pathogen-associated molecule patterns (PAMPs) [13,16,17]. Both PAMPs and DAMPs are recognized by pattern recognizing receptors (PRRs), a heterogenous group of proteins expressed on immune cells or by cells of epithelium and mesenchyme [17,18]. PRRs are members of several protein families and can be placed into extracellular fluid, bloodstream, or inside cellular compartments such as cytosol or on/in the membrane surface [19,20]. They are divided into sub-families: the nucleotide-binding oligomerization domain (NOD)-like receptors (NLR), the retinoic acid-inducible gene 1 (RIG-1)-like receptors (RLR), the C-type lectin receptors (CLRs), and the Toll-like receptors (TLRs) [18,21,22]. Multiple data showed that the Toll-like receptor 9 (TLR9), the NLR family pyrin domain containing 3 (NLRP3) inflammasome, and the stimulator of interferon genes (STING) are the mediators of the inflammation triggered by mtDNA [13]. Studies about the inflammatogenic role of mtDNA began two decades ago, when it was described that the intra-articular injection of murine and human mtDNA provokes arthritis in mice, in contrast to the administration of nuclear DNA (nDNA) [23].

### 2.2. Receptors and Pathways Activated by mtDNA

#### 2.2.1. mtDNA and Stimulation of TLR9

**Figure 1:**
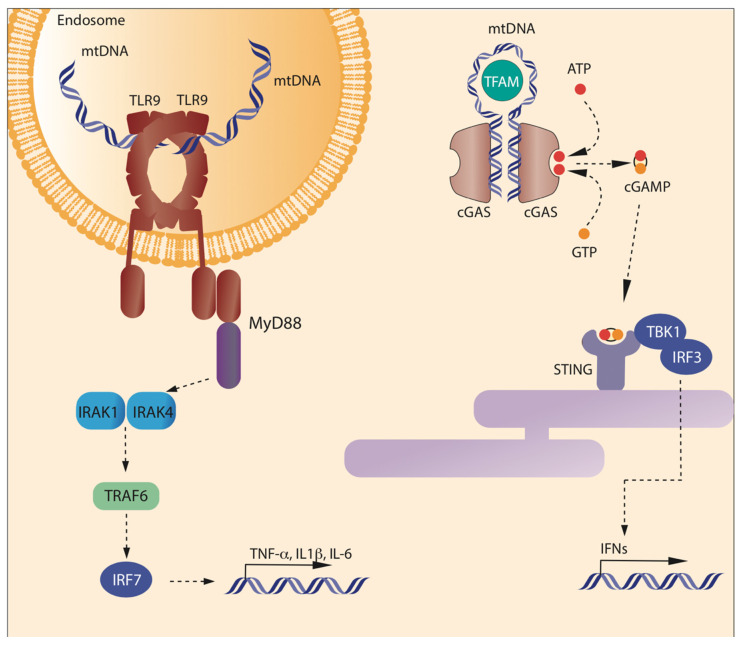
Binding of mtDNA to TLR9 and cGAS. Left: Toll-like receptor 9 (TLR9) is expressed in the inner face of the endosome membranes, as a homodimeric complex. TLR9 is activated by unmethylated CpG sequences present in DNA molecules, including mtDNA, through a sequence specific binding to the N-term of the C-shaped leucine-rich repeat region of TLR9. Each monomer binds different DNA molecules. Once bound to DNA, the cytosolic domain of TLR9 promotes the activation of MyD88 pathway, which ultimately leads to the transcription of inflammatory cytokines. In the cytosol, mtDNA can be bound by cyclic GMP-AMP Synthase (cGAS) that forms form cyclic GMP-AMP (cGAMP) from GTP and ATP. cGAMP binds to Stimulator of Interferon Genes (STING) on the endoplasmic reticulum. STING promotes the phosphorylation of IRF3 mediated by TBK1, which leads to the transcription of inflammatory genes.

TLR9 represents the first discovered receptor capable of recognizing unmethylated CpG DNA, typical of bacterial DNA and mtDNA (Figure 1) [24,25]. As with all the TLRs, TLR9 is a type I integral membrane protein, with an N-terminal ligand recognition domain, a single transmembrane helix, and a C-terminal cytoplasmic signaling domain [26]. The N-terminal domain presents the typical horseshoe-shaped structure, built from the repetition of leucine-rich repeat (LRR) motifs [27]. TLR9 is mainly localized in plasmacytoid dendritic cells, monocytes, B cells, and macrophages [22], residing in the endoplasmic reticulum (ER) and translocating to endosome after stimulation by hypomethylated CpG islands [13,22]. TLR9 and CpG-DNA form a symmetric complex with 2:2 stoichiometry. A systematic analysis of different oligonucleotides has shown that the consensus DNA sequence bound by TLR9 is TCG[T]_6–10_CG[T]_9–19_ and a total length of at least 21 nucleotides; the molecule bound is a single-stranded DNA (ssDNA), and the formation of double-strands reduces, but does not abolish, the interaction with the receptor [28]. The molecular interaction between mtDNA and TLR9 has been clarified in 2015, when Ohto and colleagues showed that the site of interaction between DNA molecules including CpG motif and TLR9 localizes in the groove at the N-terminal side of one of the TLR9 protomer. Another protomer of TLR9 recognizes the phosphate backbone of CpG motif from the opposite side, thus determining the dimerization of TLR9. In addition, it has been demonstrated that TLR9 encompasses other DNA binding sites able to make its activation and dimerization more efficient [29,30,31,32].

After the binding with DNA, TLR9 activates an inflammatory response via mitogen-activated protein kinase (MAPK) and nuclear factor kappa-light-chain-enhancer of activated B cells (NF-kB), through the interaction with myeloid differentiation primary response 88 (MYD88) adaptor protein [22]. The ability of mtDNA to activate an inflammatory response via TLR9 was described for the first time a decade ago, when it was demonstrated that mtDNA/TLR9 could activate polymorphonuclear neutrophils (PMNs) p38 MAPK [33,34]. Since then, interaction between mtDNA and TLR9 has been shown to trigger inflammatory phenotype in numerous cell types, such as plasmacytoid dendritic cells (pDCs), B and natural killer (NK) cells. In pDCs, the interaction between mtDNA and TLR9 occurs when mtDNA is liberated in association with TFAM by necrotic cells. After the release, mtDNA binds TLR9, while TFAM, in a way similar to high mobility group box 1 (HMGB1) DNA binding protein, can be associated with receptors for advanced glycation end products (RAGE), promoting type I interferon (IFN) production [35]. To a lesser extent, the same phenomenon has been described for B and NK cells. When mtDNA avoids autophagy, its accumulation increases mortality and myocarditis following treatment with pressure overload in transgenic mice, leading to a cardiac-specific ablation of lysosomal deoxyribonuclease (DNase) II. An attenuation of cardiac dysfunction has been described in the same mice, leading to a deletion of TLR9 or administration of inhibitory oligodeoxynucleotides of TLR9 and in wild type DNase II TLR9-deficient mice [36]. Stimulation of TLR9/NF-kB pathway by mtDNA and consequent inflammation response has also been described during acute lung injury after a hip fracture in young and old rats [37]. High levels of mtDNA with the capacity to activate TLR9 have been found in plasma of patients and mice with nonalcoholic steatohepatitis (NASH), driving the inflammatory phenotypes peculiar to this widespread liver disease [38].

A point that still needs to be clarified is what form of mtDNA has physiological access to TLR9. mtDNA has been undoubtedly shown to cause sterile inflammation in a TLR9-dependent manner when present in a free form, i.e., not bound to protein or encapsulated in vesicles [39,40,41]. However, the fact that most of the circulating mtDNA is not present as a free molecule, and that it has to be phagocytosed to gain access to TLR9 makes it difficult to understand the relative contribution of mtDNA/TLR9 pathway to inflammation under physiological conditions.

#### 2.2.2. mtDNA and Activation of NLRP3 Inflammasome

NLRP3 inflammasomes are multiprotein cytoplasmatic complexes of the innate immune system. They have been formed by NLRP3 sensor protein PRR that interacts with apoptosis-associated speck-like protein containing a caspase-recruitment domain (ASC) adaptor molecule to recruit the precursor form of caspase-1. Inflammasome allows the activation of caspase-1, and triggers the maturation and secretion of pro-inflammatory cytokines IL-1β/IL-18 [42,43]. NLRP3 inflammasomes are generally located in neutrophils, monocytes, dendritic cells, macrophages, and many non-hematopoietic cells [44,45,46].

The interaction between mtDNA and NLRP3 was described in mouse macrophages deficient of autophagic proteins LC3B and beclin1, and in mouse autophagy protein-deficient bone marrow-derived macrophages (BMDMs) after stimulation with lipopolysaccharide (LPS) and ATP. Deficiency of LC3B and beclin1 provokes an accumulation of dysfunctional mitochondria and the NRLP3/ROS-dependent translocation of mtDNA to cytosol. Cytosolic mtDNA participates in the secretion of IL-1β/IL-18, thus to the inflammatory response [47].

A possible role of mitochondria in the activation of NLRP3 inflammasome was proposed for the first time ten years ago, when it was shown that the blockade of mitophagy causes the accumulation of damaged mitochondria, producing high levels of ROS which in turn activate inflammasome. However, the link between ROS and NLRP3 in this model remained elusive, until the direct demonstration that NLRP3 can be bound by oxidized (ox)-mtDNA released during apoptosis. A series of seminal studies further proved the role of ox-mtDNA: it has been shown that autophagic elimination of damaged mitochondria prevents inflammasome activation [48]; that inhibition genetic of pharmacological inhibition of mtDNA synthesis prevents NLRP3 inflammasome activation; that the new synthesis, induced after the engagement of Toll-like receptors, is crucial for NLRP3 signaling; and that re-introduction of oxidized DNA into macrophages restores NLRP3 activation in models where mtDNA synthesis were inhibited. As re-introduced oxidized DNA can activate NLRP3 regardless of its origin, it is likely that NLRP3 senses the presence of 8-OH-dG within ox-mtDNA. Ward et al. reported an increased secretion of ox-mtDNA, following inflammasome activation in an in vitro cancer model [49].

#### 2.2.3. mtDNA and cGAS-STING

mtDNA can also stimulate an inflammatory response when interacting with GMP-AMP synthase-stimulator of interferon genes (cGAS-STING) pathway. cGAS is a cytosolic protein with the canonical structure of the cGAS/DncV-like nucleotidyltransferase (CD-NTase) family members: a cage-like architecture creates a deep pocket where the enzymatic site is located [50,51]. On the opposite side, a long α-helix contacts DNA. The minimal active complex is a 2:2 unit, i.e., two copies of cGAS bind two DNA helices (Figure 1), but usually an oligomeric complex is formed, where multiple copies of cGAS bind DNA to form a protein–DNA ladder. Proteins able to bend DNA and to create U-turns, such as TFAM, pre-arrange DNA in the correct position to form multimers [52]. cGAS acts as a sensor of misplaced DNA, as it binds microbial or self (nuclear or mitochondrial) DNA in the cytosol. When misplaced DNA is bound, cGAS catalyzes the production of 2′3′ cyclic GMP–AMP (cGAMP). cGAMP acts as a second messenger, by binding STING and finally mediating the transcription of IFN stimulatory genes (ISGs) and type I IFN via the phosphorylation of TANK-binding kinase 1 (TBK1) and of transcription factor interferon regulatory factor 3 (IRF3) [21,22,53].

Interaction of mtDNA with cGAS-STING signaling axis has been described in multiple conditions, which share the final leakage of mtDNA from mitochondria to the cytosol. During caspase-inhibited apoptosis, releasing of mtDNA in a BAX/BAK dependent manner, and the subsequent interaction with cGAS-STING signaling axis, was observed in mouse embryonic fibroblasts (MEFs) [54,55]. Using MEFs from transgenic mouse models, it has been demonstrated that TFAM deficiency causes the escape of mtDNA into the cytosol where it binds cGAS, triggering the STING-TBK1-IRF3 signaling pathway, finally eliciting ISG expression and increasing type I IFN response [56].

The mechanism(s) regulating the activation of the mtDNA/cGAS-STING axis has been only partially elucidated, by a combination of genetic and pharmacological approaches, and by the hints provided by diseases where an excessive production of type IFNs were observed. The analysis of cCAS-STING activity in the pathogenesis of amyotrophic lateral sclerosis (ALS) has shown the role of transactive response DNA-binding protein ~43 kDa (TDP43), one of the proteins causatives of ALS, in the cytosolic release of mtDNA. TDP43 is an RNA binding protein that plays multiple roles on RNA metabolism [57]. TDP43, normally present in the cytosol and in the nucleus, can also be present in the mitochondria, where it is likely involved in the regulation of tRNA synthesis. When present in excess in the organelle, TDP43 triggers mtDNA release into the cytosol through the opening of the mitochondrial permeability transition pore (mPTP), to activate cGAS-STING signaling and type I IFN production. Accordingly, genetic deletion of STING mitigates the disease in a mouse model of ALS [58]. These observations explain, at least in part, why inflammatory responses and excessive type I IFN production are usually observed in ALS [59]. mtDNA release to the cytosol and activation of cGAS-STING pathway can also be mediated by cellular pyrimidine imbalance [60]. A crucial player in this process is YME1L, an i-AAA protease required for efficient de novo pyrimidine synthesis. By using different cellular and animal models deprived of YME1L, or by pharmacological inhibition of pyrimidine biosynthesis, Sprenger and colleagues have recently shown that the impairment of pyrimidine cellular pool synthesis deregulates mitochondrial nucleotide uptake and mtDNA replication, and triggers mtDNA release into the cytosol with a process that requires voltage-dependent anion channels (VDAC) oligomers [60]. Interestingly, this regulatory mechanism has also been proven in *Saccharomyces cerevisiae*, suggesting that it represents an evolutionary conserved regulatory circuit that was still present in organisms that did not display inflammatory responses. Is it likely that the original function of this mechanism was to replenish the nucleotide pool in the cytosol, and that it has then been adapted to a new immunological function after the appearance of cytosolic DNA sensors.

Besides ALS, several other diseases have shown an impairment in mtDNA/cGAS-STING axis and provided further details on the mechanism underpinning the regulation of these pathways. Kerur et al. reported a contribution of mitochondrial dysfunction and cGAS signaling to the progression of age-related macular degeneration. This pathology is characterized by retinal pigmented epithelium (RPE) death, inflammasome activation, and a deficiency in the DICER1 protein, which leads to increased levels of *Alu*-retroelement RNA. However, what triggers the activation of the inflammasome remains elusive. This group demonstrated that, ultimately, this activation was dependent on the cGAS pathway. Additionally, they proved that decreased levels of DICER1 or accumulation of *Alu*-RNA resulted in the release of mtDNA into the cytosol, contributing to cGAS activation [61]. Deficiency or inhibition the ataxia telangiectasia mutated (ATM) protein can also lead to mtDNA leakage and cGAS-STING activation via an indirect mechanism: when ATM is absent or inactive, a downregulation of TFAM occurs, which favors mtDNA leakage from mitochondria [62].

By analyzing two patients with a different phenotype but a common enhanced expression of ISGs, Lepelley and coworkers have recently identified the mitochondrial membrane protein ATPase family AAA domain-containing protein 3A (ATAD3A) as a player in the regulation of mtDNA release from mitochondria and cGAS activation [63]. Indeed, dominant negative mutations of ATAD3A or knockdown of the gene resulted in increased activation of cGAS pathway. Such upregulation is mtDNA-dependent, as depletion of mtDNA in different cellular models abrogates this effect. Thus, it is likely that ATAD3A is involved in the release of mtDNA from mitochondria.

Another possible actor in the escape of mtDNA from the organelle to activate cGAS-STING is VPS13C, a protein whose mutations cause early onset, autosomal recessive Parkinson’s disease (PD). Mutations of the yeast homolog of VPS13C, Vps13, were shown to cause escape of mtDNA from mitochondria to the nucleus almost 20 years ago [64]. The silencing of VPS13C causes the activation of STING, in a mechanism that involves the combination of high mtDNA levels in the cytosol and a defect in the lysosomal degradation of activated STING [65]. Despite the exact mechanisms linking VPS13C and mtDNA remaining elusive, it is likely that a disruption of lysosome function, associated with VPS13C defects, can lead to mitochondria dysfunction and mtDNA escape caused by defective mitophagy, as observed in other models [36,66].

Finally, it has been shown that the microtubule destabilizer eribulin, a chemotherapeutic drug used in the treatment of triple-negative breast cancer (TNBC) is able to induce the production of IFN-β, independently from its antimitotic action, by causing the release of mtDNA from the mitochondria into the cytosol and the activation of cGAS [67].

## 3. Mechanisms of Release of mtDNA in Biological Fluids

**Figure 2:**
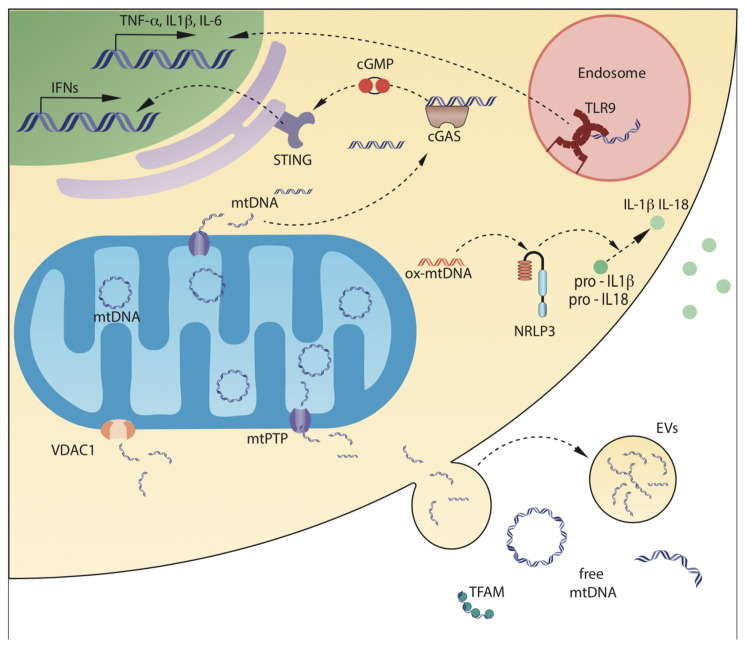
Mechanisms of mtDNA release from mitochondria, and of mtDNA sensing. mtDNA can be released by mitochondria into the cytosol, or in the extracellular space, either as circular molecules, DNA fragments, or DNA associated with mitochondrial proteins. Leakage from mDNA can be mediated by mitochondrial Permeability Transition Pore (mPTP), that causes swelling of the mitochondrial inner chamber and loss of impermeability of the Mitochondrial Inner Membrane (MIM), and by pores on the Mitochondrial Outer Membrane (MOM), formed by pro-apoptotic protein BAX or by the oligomers of the mitochondrial porin voltage-dependent anion-selective channel 1 (VDAC1). Once in the cytosol, mtDNA can interact with cGAS, which activates a pathway leading to enhanced transcription of type 1 interferons, or with NLRP3, particularly when oxidized. NLRP3 inflammasomes activate IL-1β and IL-18 by post-translational cleavage. Cells can also take up mtDNA from surrounding environment by phagocytosis; once in the endosomes, mtDNA can interact with the Toll-like receptor 9, which activates a pathway leading to transcription of pro-inflammatory cytokines TNF-α, IL-1β, and IL-6. Finally, mtDNA can be released outside the cells either by passive release, mediated by rupture of plasma cell membrane integrity, or by active release through extracellular vesicles.

mtDNA is normally confined within the mitochondrial matrix, packed in nucleoids [68], and the presence of mtDNA in the cytosol or outside the cells is a consequence of the loss of mitochondrial integrity. Although this phenomenon has been largely investigated in the last years, little is known about the molecular mechanisms triggering the release of mitochondrial genome within the extracellular space. As it will be reviewed in the following sections, mtDNA can be released in a passive or accidental pathway, triggered by cell necrosis or apoptosis, or by an active or regulated process, mediated by specific mechanisms (Figure 2).

### 3.1. Release of mtDNA from Mitochondria to the Cytosol

The disruption of mitochondrial integrity and the following release of mitochondrial content into the cytosol has been a topic of increasing interest over the last years, and the mechanisms leading to the release of mitochondrial content have been partially elucidated.

#### 3.1.1. mtDNA Release Mediated by Mitochondrial Outer Membrane Permeabilization (MOMP)

The mitochondrial outer membrane permeabilization (MOMP) is the driving process that leads to caspase activation in intrinsic apoptosis [69]. MOMP is a highly regulated process, controlled through the interactions of the members of the B cell lymphoma (BCL-2) family that leads to the release of intermembrane proteins into the cytosol [69,70]. The process initiates with the permeabilization of the mitochondrial outer membrane (MOM), mediated by the formation of macropores, formed by BAX/BAK oligomers. These BAX/BAK macropores allow the herniation of the inner mitochondrial membrane (IMM), resulting in the loss of membrane integrity and the subsequent release of the mitochondrial content, including mtDNA, into the cytoplasm [71,72]. This process is believed to be a common mechanism in physiological conditions, occurring in any cell controlled by BAX/BAK, and it ends with the release of cytochrome c and the activation of caspase cascades [72].

Interestingly, the activation of the cGAS/STING pathway by mtDNA can determine the activation of a caspase-independent cell death (CICD) mechanism, under conditions of caspase inhibition [72,73]. As the IMM was thought to conserve its integrity during apoptosis, the mechanism allowing the leak of mitochondrial genome in the cytosol during CICD was not clear. This paradox was explained in recent studies. Riley et al. and McArthur et al. demonstrated that the IMM could undergo permeabilization, resulting in the release of mtDNA into the cytosol, where it is able to bind with and activate the cGAS-STING pathway [72,73].

Another mechanism of mtDNA release, mediated by a BAX/BAK-independent MOMP pathway, was reported recently by Kim and colleagues. They described the formation of MOM pores and the following MOMP, mediated by VDAC in MEFs [74]. This was the first time this event was observed in live, non-apoptotic cells. VDAC, also known as mitochondrial porin, is a major transport protein of the OMM, and mediates the transport of anions, cations, ATP, Ca^2+^, and metabolites across the membrane via a voltage-sensing mechanism [75]. In humans, three different isoforms have been described, namely VDAC-1, VDAC-2, and VDAC-3, each of them with different properties and functions. Human VDAC-1, represented as a β-stranded barrel, appears to be the most prevalent isoform, and it is related to apoptosis [76,77]. VDAC1 can form oligomers on the MOM, and its oligomerization is coupled to the induction of apoptosis [78].

Kim et al. therefore investigated the mechanism of mtDNA release triggered by VDAC in an animal model of systemic lupus erythematosus (SLE), in conditions where there is no activation of the BAX/BAK complex [74]. The results showed that the formation of mitochondrial pores allows the release of the mitochondrial genome to the cytosol, inducing the activation of cGAS-STING pathway. Whether MOMP occurs via BAX/BAK or VDAC pathway was proposed to be mediated by the cell stress level: the latter mechanism is predominant in conditions of moderate stress, whereas the former is present in extreme stress or apoptosis. Interestingly, it was observed that the treatment with an inhibitor of VDAC oligomerization reduced the lupus-like disease in the model [74]. VDAC-1 interacts with mtDNA by means of three positively charged residues at the N-term, which binds to negatively charged mtDNA and promotes its oligomerization on MOM. The antiviral factor mitochondria-associated vaccinia virus-related kinase 2 (VRK2) turned out to be the key regulator of VDAC-1-mediated leakage of mtDNA from mitochondria. Upon viral infection, VRK2 associates with VDAC-1, and promotes its oligomerization. VRK2 appears to facilitate binding of mtDNA to VDAC-1, VRK2 deficiency impairs mtDNA binding to VDAC-1 and VDAC-1 oligomerization, while its overexpression markedly enhances the binding [79].

#### 3.1.2. mtDNA Release Mediated by Mitochondrial Permeability Transition Pore (mPTP)

The mPTP is a non-specific component of the IMM. The opening of mPTP allows the free transport of small molecules and metabolites and, in this context, the flux of protons is of great importance.

Under conditions of mitochondrial stress, mPTP opens, inducing a series of irreversible events that lead to mitochondrial dysfunction. Following mPTP opening, the flux of ions will result in calcium overload, mitochondrial depolarization, inhibition of ATP synthesis, depletion of pyridine nucleotide, and, at last, respiratory inhibition, metabolic impairment, matrix swelling, and cell death [80,81]. Therefore, mPTP can be defined as a mechanism of regulated cell death and, consequently, it would be expected to observe mPTP implication in pathologies associated with mitochondrial dysfunction, as it has been largely reported [82]. The swelling of the mitochondrial matrix is a key event, as ultimately it will lead to the rupture of the OMM, inducing the release of proapoptotic proteins [80,81]. Besides allowing the efflux of metabolites, mPTP constitutes a pathway for mtDNA release. Garcia et al. reported that under oxidative stress, mtDNA was released through mPTP in rat liver cells. In this model, mitochondria were stimulated with Fe^2+^, H_2_O_2_, and calcium ions to induce oxidative stress. As a consequence, mitochondria swallowing and hydrolyzation was observed, together with the formation of thiobarbituric acid-reactive substances, a marker of oxidative stress [83].

Despite the extensive research focused on the understanding of the release of mtDNA mediated by mPTP, a definitive explanation on the mechanism(s) underpinning its release has not been provided to date. In particular, it is not known if the leak of mtDNA is a completely passive process. Based on the works previously mentioned, it is likely that, when an external damage triggers the opening of mPTP, the inner mitochondrial components escape, including the mitochondrial genome. However, the relationship between mPTP opening and the presence of extracellular mtDNA—often observed after an external damage—is not clear.

### 3.2. Mechanisms of Release of mtDNA outside the Cells

The presence of mtDNA has been observed in different biological fluids, including plasma and serum [84,85], cerebrospinal fluid (CSF) [84], or synovial fluid [23,86], and there is an increasing interest in mtDNA and cell-free mtDNA (cf-mtDNA) as potential biomarkers of inflammation and predictors of mortality. The levels of mtDNA in vivo are usually measured in blood, as it is relatively easy to obtain and analyze. However, the exact source of mtDNA remains unknown in most cases. Several lines of evidence indicate that more than 90% of cf-mtDNA is actually present in intact, circulating mitochondria, whose number has been estimated to be around 10^5^–10^6^/mL [87,88]. These mitochondria are normally present in healthy subjects, clearly indicating that most circulating mtDNA does not have any inflammatory effect, and further confirms that only free mtDNA is able to cause inflammation [87,89].

mtDNA can be present in extracellular fluids due to an active and tightly regulated process, as in the case of release by neutrophils (NETosis) or other leukocytes, or as a consequence of passive release from dead cells. Although both mechanisms are pathophysiologically relevant, the relative contribution of active vs. passive release is still a matter of debate.

#### 3.2.1. Active Release of mtDNA: Extracellular Traps (ETs)

One of the best characterized sources of extracellular mtDNA are neutrophils. In response to bacterial PAMPs, neutrophils release highly organized, web-like structures composed of decondensed chromatin, cytosolic and granule proteins called neutrophils extracellular traps (NETs) [90]. Neutrophil extracellular traps kill bacteria. The majority of DNA present in NETs is of nuclear origin, but mtDNA is also present [91] The presence of mtDNA is not incidental, and has an inflammatogenic potential [91,92]. The formation of NETs has been linked to the pathogenesis of several inflammatory diseases, including trauma [93,94], diabetes [95], or NASH [96].

Two types of NETosis may occur: suicidal NETosis and vital NETosis. Although considered a misnomer by the Cell Death Nomenclature Committee, which does not recommend the use of the word “NETosis” when not associated with cell death [67], the term “vital NETosis” is still very common and used in this review for clarity. Suicidal NETosis is characterized by the disruption of plasmid and nuclear membrane, in a process lasting 5–8 h [97]. After the activation of neutrophils, calcium is released from the ER into the cytoplasm, ultimately triggering the generation of NADPH-depended ROS [46,47,53]. ROS elicit nuclear and granule breaking, allowing the mixing of their content [47,53]. Myeloperoxidase (MPO) and neutrophil elastase (NE), enzymes located in azurophilic granules and peptidyl arginine deaminase 4 (PAD4) generate the decondensation of chromatin [53,55] and, finally, the rupture of the plasma membrane allows the release of NETs outside the cell [47,53]. Conversely, vital NETosis is a very fast (5–60 min) process that does not involve the death of neutrophils, which remain intact and functional [53]. During vital NETosis, neutrophils maintain the integrity of the structure of the plasma and nuclear membrane [18,98]. In this type of NETosis, NETs formation was found to be triggered by ROS and NETs. A form of vital NETosis, where ETs contained DNA exclusively from mitochondrial origin, was first observed in 2009 [92]. The inhibition of mtDNA release was observed after the treatment of neutrophils with diphenyleneiodonium (DPI), an inhibitor of ROS. Moreover, it was observed that ROS-deficient neutrophils did not release mtDNA. All these findings proved that the formation of NETs and the following mtDNA release is not dependent on cell death, but on the presence of ROS [92]. McIlroy et al. reported the formation of NETs containing mtDNA in trauma patients. In accordance with the results reported by Yousefi et al. [92], the treatment with DPI resulted in the inhibition of NETs formation, with the subsequent blocking of mtDNA release [93]. The inhibitory effect of DPI on NETs formation was confirmed in another study, where an increased release of mtDNA was observed in trauma, which triggered the formation of NETs. The attributed mechanism was dependent on TLR9 activation. Interestingly, it was found that NETs formation was dependent on age, as NETs levels in elderly trauma patients were observed to be lower than in younger patients [94]. The formation of NETs appears strictly intertwined with the activation of the intracellular pathways triggered by mtDNA. For instance, the presence of increased circulating levels of cf-mtDNA has been observed in patients with sickle cell disease (SCD). The increase in cf-mtDNA levels was suggested to trigger the activation of NETs formation and cGAS-STING pathway. The authors reported mitochondrial retention by circulating SCD red blood cells and suggested that this could be the source of the elevated levels of cf-mtDNA [99]. Two mechanisms have been proposed to explain how mtDNA is released in the extracellular space. In the first scenario, mtDNA is first released into the cytosol, and then is enclosed in vesicles that fuse with the cell membrane, leading to mtDNA extrusion. An alternative possibility is that a fusion of mitochondrial and cell membrane occurs, leading to the release of mitochondrial content, including mtDNA, in the extracellular space [100].

Interestingly, the removal of damaged mitochondria in human neutrophils does not take place through mitophagy, as it occurs in most cells, but a different pathway is activated [101]. In healthy neutrophils, the mitochondrial components are extruded into the extracellular space. Conversely, ox-mtDNA is removed through a different mechanism, mediated by the disassembly of mtDNA/TFAM complexes, followed by the formation of vesicles where ox-mtDNA is enclosed, and their exportation into lysosomes for degradation. This phenomenon has important pathological implications: in SLE, neutrophils exhibit a defect in this mechanism, leading to an inefficient removal of ox-mtDNA, which in turn accumulates inside the mitochondria and is finally released as ox-mtDNA bound to TFAM [101]. As ox-mtDNA is particularly efficient to activate pDC, it leads to massive production of type I IFNs.

Like neutrophils, eosinophils can produce DNA-based extracellular traps (comprising of anti-microbial peptides, histones, and DNA) to capture and kill pathogens. The first study demonstrating the capability of eosinophils to produce extracellular traps showed that cells remain viable after trap formation, and that the DNA released was mtDNA. The mitochondrial origin of the leaked DNA was recognized by a combination of molecular biological and microscopic techniques [102]. In analogy with the phenomenon described in neutrophils, these structures were named eosinophils extracellular traps (EET). The extracellular trap recognizes a microbial pathogen, restricts their mobilization, and is responsible for their death. Enzymatic degradation of extracellular DNA results in the incapability of eosinophils to kill bacterial pathogens in the extracellular space [103]. The mitochondrial origin of EET has not been conclusively confirmed, and an eventual study has shown the presence of nuclear DNA, rather than mtDNA, in the EET [104]. Therefore, although DNA in the extracellular traps is unlikely to be highly toxic for bacterial pathogens, it is indispensable for the anti-bacterial impacts mediated by eosinophils. The eosinophil extracellular traps process also depends on the production of ROS, such as neutrophil and mast cells extracellular traps. mtDNA release was also observed in B, T, and NK cells in response to oligodeoxynucleotides [105].

The functional role of mtDNA traps is not completely clear. Leukocytes, and in particular eosinophils and lymphocytes, contain a few mitochondria and a low amount of mtDNA [106,107] casting doubts on the possibility that ETs made exclusively of mtDNA can be functional in physiological conditions. Furthermore, in the case of lymphocytes, extracellular mtDNA released by lymphocytes is not associated with lytic enzyme, suggesting that it has exclusively a proinflammatory function [103,108].

#### 3.2.2. Active Release of mtDNA Mediated by Extracellular Vesicles (EVs)

A second active source of extracellular mtDNA are extracellular vesicles (EVs), a group of membrane bound particles with a heterogeneous origin and composition released into body fluids, used for cell-to-cell communication [109,110]. Although classification of EVs is still controversial, they are conventionally divided in three major populations: exosomes, microvesicles (MVs) [111], and apoptotic bodies (ABs) [112,113]. Exosomes are intracellularly generated EVs whose dimensions range from 30 to 150 nm, with lipid bilayer structures released by cells to transfer small molecules to other cells; they can contain nucleic acids and proteins derived from within cells [114]. Microvesicles are EVs released from plasma membranes, whose dimensions can be up to 1 μm [115]. ABs are the final product of programmed cell death, and their dimensions range between 1–5 μm. Initially, it was thought that the release of EVs was merely a mechanism of eliminating cellular waste components. However, now it is known that EVs have an important role in intercellular communication, acting as transport cargo of different messengers, including DNA, RNA, and proteins [116], and that their release is an active, tightly regulated mechanism. In the clinical field, extracellular vesicles are gaining attention as promising candidates for diagnostic and therapeutic tools. Many host factors have been shown to be involved in the secretion of EVs [117], including the Rab GTPase family Rab27 and Rab35 [118,119].

Mitochondrial components, including mtDNA are common cargo of EVs [120,121,122,123,124,125]. The packaging of mtDNA in microvesicles as an alternative pathway of release of mtDNA has been described years ago, when Guescini and co-workers demonstrated the migration of mitochondrial genomic material in in vitro models using astrocytes, myoblasts, and glioblastoma cells [126,127]. Then, Sansone and colleagues confirmed the release of mtDNA via EVs in stromal cells and the subsequent transfer to cancer cells in a model of breast cancer. The mechanism by which the mitochondrial genomic material was packed into EVs was not clarified, but the group suggested that the transfer of mtDNA occurs as a defense mechanism of the malignant cells to preserve the metabolic activity, eluding the metabolic dormancy induced by therapy [128]. The packaging of mtDNA into vesicles was further demonstrated in an in vitro placental cell culture. Interestingly, EVs levels of mtDNA were increased upon exposure to anti-phospholipid antibodies (aPL), which are known to increase the risk of preeclampsia. Although no mechanistic explanation was provided, the authors suggested that the treatment with aPL results in mitochondrial rupture, with the subsequent release of mtDNA, which could be packed into EVs. Additionally, it was also observed that these placental vesicles induced the activation of endothelial cells through a TLR9 mediated pathway [129]. An increased release of microparticles containing mtDNA was also observed in alcoholic neutrophilia. In this model, the increase in circulating mtDNA-containing microparticles triggered the activation of neutrophils, contributing in this way to the pathogenesis of alcohol-induced liver injury. This statement was demonstrated with animal models, in which the treatment with mtDNA-enriched microparticles triggered the generation of neutrophilia. The authors attributed this finding to the activation of the TLR9 receptor, as the effect described previously was not observed in animals that presented a deficiency for TLR9 [130]. Ye et al. described increased levels of exosomes carrying mtDNA in the plasma of patients with chronic heart failure. It was observed that the exosomes were internalized by cells, triggering an inflammatory response, which induced the secretion of the proinflammatory cytokines IL-1β and IL-8, and activating the TLR9-NF-κB pathway. The extent of the inflammatory response was observed to be tightly dependent on the mtDNA copy number, and the inflammatory response was observed to be inhibited after the treatment with an inhibitor of TLR9 [131].

Eventual studies demonstrated that mitochondrial cargo within EVs can be released by different cell types in response to proinflammatory stimuli [47,132,133,134,135,136]. It must be noted, however, that mitochondrial proteins have been also detected in EVs in the absence of any proinflammatory stimuli [124,137]. The functional role of this mitochondrial cargo is still debated, but some studies suggested that the packaging of mtDNA in EVs can represent one of the mechanisms of mitochondrial transfer that can be used for mitochondrial maintenance, or for rescuing compromised mitochondrial function in various physiopathological conditions [121,125,138,139,140].

Only recently the factors regulating trafficking of mitochondrial components, including mtDNA, into EVs have been identified [141]. Mitochondria can produce their own vesicles, named mitochondria-derived vesicles (MDVs), to transport mitochondrial components to nearby organelles. MDVs were previously known for their role mitochondrial antigen presentation, but Todkar and colleagues showed that they are also responsible for transportation of mitochondrial components to EVs, in a process that depends on optic atrophy 1 (OPA1) and sorting nexin 9 (Snx9) proteins. In the presence of damaged mitochondria, which potentially contain more inflammatogenic molecules, Parkin inhibits this pathway and targets MDVs for lysosomal degradation, thus preventing release of DAMPs [141].

#### 3.2.3. Passive Release of mtDNA

“Passive” release of mtDNA is mainly observed due to cell death, by apoptosis of necrosis. Although “passive” release is commonly used to describe mtDNA release following cell death, it must be underlined that this process is not entirely uncontrolled, and different factors can modulate the presence of mitochondria in cell debris, or their release in the surrounding environment during cell death.

During apoptosis, fragmentation of cells in ABs allow efficient clearance of apoptotic cells and prevent unwanted immune response versus self-antigens. As stated above, apoptotic bodies are large EVs, and contain DNA, RNA, and proteins, including mitochondrial proteins and mtDNA. Contrary to previous beliefs, formation of ABs is a tightly regulated process controlled by different factors, and in particular by the kinase Rho-associated, coiled-coil-containing protein kinase 1 (ROCK1) and the membrane channel pannexin 1 (PANX1) [142]. The material contained in ABs is used in some models used for cell-to-cell communication [143,144,145]. ABs are heterogeneous in nature, and some of them, but not all, contain intact but dysfunctional mitochondria [146]. As ABs share the surface markers with their cell of origins [147], these markers can be potentially used to determine the origin of ABs, and consequently of mitochondria and mtDNA found within them.

Nevertheless, apoptotic cells can—at least in some models—actively secrete intact mitochondria, challenging the idea that the organelles are released only via ABs [132]. Interestingly, actively secreted mitochondria during apoptosis returned out to be inflammatogenic. In fact, intact mitochondria can recruit neutrophils and, when internalized by macrophages, activate inflammasome via NLRP3. Similarly, mitochondria can be actively secreted during necroptosis, a form of regulated necrosis, but do not cause inflammasome activation [132].

Passive release during necrosis has been mainly studied in pathological conditions causing acute damage, including (but not limited to) traumas [148,149,150], sepsis [151], ischemia/reperfusion [152,153], or chronic diseases, such as viral infections [154], rheumatoid arthritis, cancer [155], or neurodegenerative diseases [156]. In most of these conditions, a positive correlation has been observed between the amount of cf-mtDNA present in the bloodstream and the extent of necrosis or damage observed, indirectly suggesting that mtDNA is freely released from necrotic cells into the surrounding environment by rupture of cell membranes from mechanical trauma. Longitudinal studies, in which the concentration of cf-mtDNA were monitored post-injury, strengthened this idea [151,157].

Furthermore, the observation that mtDNA measured in extracellular environment is associated with other mitochondrial DAMPs, corroborates the idea that an uncontrolled, passive release of mitochondrial products (including mtDNA) occurs in these clinical settings, rather than a regulated and selective release of mtDNA [158]. Passive release can be observed also in conditions where no macroscopic injuries occur. Strenuous exercise induces cf-mtDNA release, whose extracellular levels increase immediately post exercise. It is also likely that, in this case, cf-mtDNA is passively released due to necrosis, but no formal proof has been provided.

### 3.3. Anatomical Source of mtDNA

The observations that damage to different organs can cause cf-mtDNA transient increase in the blood indirectly prove that multiple sources of mtDNA are present in the body. However, the primary source of extracellular mtDNA remains elusive for several reasons. From a technical point of view, it is difficult to distinguish mtDNA molecules coming from different cell types. The only way to do that is indirect, by using cell-specific molecules associated with mtDNA. Moreover, not all cell types have the same amount of mtDNA, not all cell types have the same tendency to undergo apoptosis or necrosis, and not all cell types release the same amount of EVs. Furthermore, the capability to release mtDNA or intact mitochondria has been shown ex vivo for several cell types, including (but not limited to) platelets, leukocytes, or endothelial cells [136,159,160], and in vitro for different cell lines [87,89,160,161,162,163,164]. The relative contribution of these sources has been only partially elucidated. By combining flow cytometry and proteomic approach, Stephens et al. have recently shown that circulating mitochondria present in human platelet-depleted plasma derive mainly from endothelial cells (49%), but also from platelets (11%) and leukocytes (9%). Interestingly, proteomic analysis showed that circulating mitochondria are associated with protein markers present in extracellular vesicles, suggesting that a fraction of these intact mitochondria circulates as EV cargo [88]. However, this study does not clarify the origin of free mtDNA, not associated with mitochondria, which is likely the most relevant in triggering inflammation. The main pathophysiological conditions where a release of mtDNA from the mitochondria to the cytosol and/or to the extracellular space discussed in this section are summarized in Table 1.

## 4. Concluding Remarks

As highlighted in this review, mtDNA is not simply the own genome of mitochondria, but also a key player in the regulation of several inflammatory mechanisms. The intense research in this field in the last 10 years has revealed that multiple mechanisms to sense mtDNA exist, and that they evolved to detect mtDNA from different sources—extracellular and intracellular—and in different forms, such as cf-mtDNA, as protein-bound DNA, or as part of whole mitochondria.

Although the enormous progress made in the identification of the molecular mechanisms regulating mtDNA release and sensing during inflammation, many questions remain open. First, it is not clear how mtDNA trafficking within the cell is regulated, and the precise mechanisms that allow the release of mtDNA from mitochondria. Second, it must be understood which is the physiological role of mtDNA when present as cargo in EVs, and which cells represent the main target(s) of mtDNA-mediated inflammatory signals. Third, the relative contribution of the different sources of mtDNA to the circulating amount of mtDNA remains to be better defined.

Right: cGAS recognizes cytosolic DNA, including mtDNA released from the mitochondrion—and produces cGAMP. Two binding sites are present in each cGAS monomer opposite the catalytic site. Multiple cGAS molecules bind two DNA helices to form an oligomeric structure; only two cGAS are represented in this cartoon. TFAM can promote oligomerization by bending DNA. Then, cGAS synthesizes cGAMP, which in turn will activate STING.

## Figures and Tables

**Table 1:** Role of mtDNA as inflammatory molecules in human pathologies described in this review.

Pathology		Observation/Mechanism	Type of Study/Sample	Reference
Trauma	Systemic inflammatory response syndrome (SIRS)	mtDNA plasma levels were significantly higher in trauma patients, and a correlation between mtDNA levels and clinical severity was observed	Blood from patients with SIRS	[148,149]
	Multiple organic dysfunction syndrome (MODS)	mtDNA concentrations were predictive for the development of MODS (in patients) or organ dysfunction (in an animal model)	Plasma from severely injured patients	[150] [149]
	Traumatic injury and shock	Release of mtDNA triggers the development of severe tissue injury	Plasma from an animal model of trauma	[150]
	Trauma	TLR9-mediated NETs formation triggered by mtDNA	Plasma from trauma patients	[94]
	Trauma and sepsis	An increase in mtDNA plasma levels was observed, although differences in disease course and prognostic were observed, suggesting that the mechanism of release of mtDNA is different between the two groups	Blood from patients presenting trauma or severe sepsis	[151]
	Trauma and haemorrhagic shock	mtDNA release triggers the activation of neutrophils	Rat model of trauma and haemorrhagic shock	[34]
Autoimmune origin	Multiple sclerosis (MS)	mtDNA level, together with other pro-inflammatory cytokines, was observed to be higher in patients with progressive forms of MS, which probably contributes to the systemic inflammation present in the pathology	Plasma from MS patients	[84]
		Increased levels of cf-mtDNA in patients with relapsing-remitting form of MS An inverse correlation was observed between cf-mtDNA and disease duration	CSF from MS patients	[165]
	Lupus-like disease	VDAC-mediated mtDNA release	In vitro and in vivo animal model of SLE	[74]
		Neutrophil-mediated ox-mtDNA release	Blood from SLE patients	[101]
		NETosis inductors triggered the release of ox-mtDNA, leading to STING activation	Blood from patients with CGD or SLE In vitro models Iv vivo animal model of CGD or SLE	[91]
Cancer	Advanced epithelial ovarian cancer (EOC)	The levels of mtDNA ascites were correlated with worse outcome in EOC patients	Blood from EOC patients	[155]
	Hormonal therapy-resistant breast cancer	mtDNA release via exosomes	Blood from patients with breast cancer In vitro and in vivo animal cancer models	[128]
	Myelodysplastic syndromes (MDS)	Ox-mtDNA release after inflammasome activation	Blood from patients with MDS In vitro cancer model	[49]
Other	Sickle cell disease	Increased circulating levels of cf-mtDNA Activation of NETs formation and cGAS-STING pathway triggered by elevated levels of cf-mtDNA The results showed a mitochondrial retention by circulating SCD red blood cells, therefore the authors suggested that this could be the source of the elevated levels of cf-mtDNA	Blood from SCD patients	[99]
	Non-alcoholic steatohepatitis	mtDNA activation of TLR9	Blood from patients with non-alcohol steatohepatitis In vivo models of NASH	[38]
	Macular degeneration	cGAS activation by mtDNA, released into cytosol by Alu-RNA accumulation	In vivo animal model of RPE degeneration	[61]
